# Positive and Negative Changes in Food Habits, Physical Activity Patterns, and Weight Status during COVID-19 Confinement: Associated Factors in the Chilean Population

**DOI:** 10.3390/ijerph17155431

**Published:** 2020-07-28

**Authors:** Daniela Reyes-Olavarría, Pedro Ángel Latorre-Román, Iris Paola Guzmán-Guzmán, Daniel Jerez-Mayorga, Felipe Caamaño-Navarrete, Pedro Delgado-Floody

**Affiliations:** 1 Department of Physical Education, Sport, and Recreation, Universidad de La Frontera, Temuco 4780000, Chile; daniela.reyes@ufrontera.cl; 2 Department of Didactics of Corporal Expression, University of Jaen, 27301 Jaen, Spain; platorre@ujaen.es; 3 Faculty of Chemical-Biological Sciences, Universidad Autónoma de Guerrero, Guerrero 39087, Mexico; ipguzman2@gmail.com; 4 Faculty of Rehabilitation Sciences, Universidad Andres Bello, Santiago 7591538, Chile; daniel.jerez@unab.cl; 5 Faculty of Education, Universidad Católica de Temuco, Temuco 4780000, Chile; marfel77@gmail.com

**Keywords:** lifestyle, COVID-19, physical activity, eating habits, obesity

## Abstract

The association between the changes in lifestyle during coronavirus disease 2019 (COVID-19) confinement and body weight have not been studied deeply. Therefore, the aim of the present study was to determine lifestyle changes, such as eating habits and physical activity (PA) patterns, caused by confinement during the COVID-19 pandemic and to analyze its association with changes in body weight. Seven hundred participants (women, *n* = 528 and men, *n* = 172) aged between 18–62 years old of the Chilean national territory participated in the study. Food habits, PA, body weight, and sociodemographic variables were measured through a survey in May and June 2020. The body weight increase presented positive association with the consumption of fried foods ≥ 3 times per week (OR; 3.36, *p* < 0.001), low water consumption (OR; 1.58, *p* = 0.03), and sedentary time ≥6 h/day (OR; 1.85, *p* = 0.01). Conversely, fish consumed (OR; 0.67, *p* = 0.03), active breaks (OR; 0.72, *p* = 0.04), and PA ≥ 4 times per week (OR; 0.51, *p* = 0.001) presented an inverse association with body weight increase. Daily alcohol consumption (OR; 4.77, *p* = 0.003) was associated with PA decrease. Food habits, PA, and active breaks may be protective factors for weight increase during COVID-19 confinement.

## 1. Introduction

Coronavirus disease 2019 (COVID-19) has affected many factors in all countries, including labor, economy, production, health, and consequently, lifestyle. This pandemic has led to strict decisions to control the chain of virus transmission, indicating physical distancing and a significant reduction in mobility as the primary prevention measure, calling on nations to implement quarantines and state plans that promote teleworking [1,2]. The first contagion notified in Chile was on 3 March 2020. By the middle of that month, communal quarantines began to be decreed according to indications of the Coronavirus plan implemented by the Ministry of Health. In this epidemiological panorama, Chile has accumulated up to 16 July 2020, 366,595 cases and 8580 deaths, and the country has one of the highest rates per million inhabitants in the world [2]. Although limitations on free transit and physical distance of more than 1.5 m per person are the most effective strategies to reduce COVID-19 infections, unfortunately, this brings with it a low level of physical activity (PA) of the population [3] and changes in dietary habits related to daily life patterns, even affecting food security in vulnerable populations [4]. Additionally, one area of great concern is the long-term effects of this pandemic on body weight management in adults [5].

The experience of others countries in Europe regarding the population at risk for COVID-19, has reported that those with the highest vulnerability are those with non-communicable diseases (NCD), such as obesity [6]. In this regard, Chile is leading in the number of overweight and obese people, with 74% according to the latest report from the Organization for Economic Co-operation and Development (OECD) [7]. Therefore, the study of protective factors against obesity is a priority.

Food habits can be protective factors for health and body weight increase [8]. The association of food quality and exacerbation of the clinical scenario in patients with malnutrition due to excess consumption has been presented in developing countries, identifying westernization of the diet (WD) as one of the causes. WD is characterized by high contributions of sugars and refined flours, high consumption of saturated fats, low levels of fiber, low consumption of unsaturated oils, and consequently, low contributions of micronutrients and antioxidants, which are the main regulators of metabolism and the immune system [9]. These poor habits and an unbalanced diet cause chronic activation of the innate system and an inhibition of the adaptive immune system response by increasing oxidative stress, eventually creating a delayed adaptive response as a defense against pathogens [9,10]. For this reason, recommendations indicate the improvement of eating habits through a balanced, safe, and varied diet to keep chronic diseases under control, and thus strengthen the immune system.

Action plans based on quarantine have created a situation where people are in the same place for an extended stay, promoting sedentary behaviors, such as exposure to screens (video games, computers, tablets, smartphones, television), teleworking for those people who can perform tasks from home, and the closing of sports spaces and gyms. In recent months, the pandemic has caused a reduction in energy expenditure and outdoor sports activities. International organizations have recommended PA for at least 30 min, five days a week or maintaining the recommendation of 150 min of activity per week at a moderate intensity, in addition to breaking the sedentary routine in the case of telework every one hour [11]. Failure to comply with PA recommendations leads to functional and structural deterioration of the organism, manifesting itself in decreased physical fitness, a worsening of metabolic and cardiovascular parameters, changes in body composition with decreased muscle mass and increased mass adipose, more depressive symptoms, and decreased well-being in general, among others [12]. Recent research (37,252 French adults) reported unfavorable changes such as body weight increase (for 35%; +1.8 kg on average), decreased PA (53%), increased sedentary time (63%), and unhealthy food habits during COVID-19 confinement [13].

Because of these concerns, maintaining eating habits and PA during the COVID-19 pandemic is necessary to protect metabolic adaptations, reduce systematic inflammation, and improve nutritional behaviors that can mitigate the effects of confinement. Therefore, the aim of the present study was to determine lifestyle changes, such as eating habits and PA patterns, caused by confinement during the COVID-19 pandemic and to analyze its association with changes in body weight and physical status.

## 2. Materials and Methods

### 2.1. Participants

This study was cross-sectional and based on a voluntary sample for convenience. There were 700 participants in the Chilean national territory, including 172 men and 528 women (24.6% and 75.4%, respectively) aged 18–62 years. Inclusion criteria were: (a) age between 18–62 years old; (b) be Chilean or foreigners residing in Chile, and (c) have internet access. Participants were excluded if they presented: (a) unable to give consent, (b) intellectual limitations, or (c) no internet access. The study was completed in accordance with the Declaration of Helsinki (2013) and was approved by the Department of Physical Education, Universidad de La Frontera, Chile (project number DFP20-0032).

### 2.2. Self-Report Measures

The information was collected by an electronic survey designed by a multidisciplinary team uploaded to the Google forms platform. This instrument was piloted with 20 people to evaluate any unforeseen problems in question design and response collection (i.e., in April 2020). Once the survey was tested and validated by the team, it was shared by institutional emails, Facebook, Instagram, WhatsApp, and Twitter in May and June 2020 (i.e., for eight weeks). The survey was divided into four sections, including informed consent, personal history, food habits, and physical activity patterns.

#### 2.2.1. Study Presentation and Informed Consent

The first section presented the details of the research and informed consent. Participants were asked to be as honest as possible and to report reality in all areas faithfully. The responses were anonymous and confidential, without reporting the name or any personal information. Participants were free to leave the survey at any stage before the submission process. Responses were only accepted and considered in data processing by the research team when “submit” was selected.

#### 2.2.2. Personal History

The second section was related to personal background: sex (female, male), age (years), education level (primary, secondary, university, technical education, postgraduate), number of people who shared the same place for quarantine, socioeconomic level (low, middle-low, middle, middle-high, high), current occupation (medical leave, distance education, telework, unpaid domestic work, retired, blended work, unemployed, normal shift, independent work), marital status (single, married, common law married, separated, widowed), body weight (kg), and size (m). The body mass index (kg/m^2^) and its classification category (normal weight, overweight, or obese) were calculated. In addition, they were asked for information on body weight before and after confinement (no change, increase, or decrease).

#### 2.2.3. Food Habits

For the collection of antecedents related to eating, a daily and weekly consumption frequency survey was used. This tool consisted of gathering as much information about the frequency of weekly and daily beverage consumption (1, 2, 3, 4, 5, 6, and 7 times a week or does not consume) and food daily (1, 2, 3 or more times a day, or does not consume) from each food group (cereals, legumes, dairy products, red meats (beef, lamb, pork), white meat (chicken, turkey), fish, fruits, vegetables, water, and alcohol. Participants were consulted to determine unhealthy foods and the types of preparations or culinary techniques used, according to “The Dietary Guideline of the Chilean Population” (DGCP) [14]. Regarding behavioral changes compared to before COVID-19 confinement, questions were asked about increasing, maintaining, or decreasing diet in general or if cooking was performed more than before, less than before, or was maintained. Additionally, the general perception of diet was consulted, as to whether it was healthier, less healthy, or maintained compared to before COVID-19 confinement.

#### 2.2.4. Physical Activity Patterns

Regarding PA, we asked about the frequency of PA per week (1, 2, 3, 4, 5, more than 6 times a week, or do not do it) and the number of minutes dedicated per session according to the current references of PA for the population over 18 years [11]. They were asked what type of PA they performed (combined or mixed, yoga or Pilates, aerobics or jogging, calisthenics, resistance training) and if they had checked social networks to find exercise routines (yes, no). Sedentary behavior was reported in hours per day when sitting or lying down, not counting sleep, and the presence of active breaks in the case of teleworking (yes, no).

### 2.3. Data Analyses

Statistical analyses were performed using STATA V.13.0. (StataCorp, College Station, TX, USA). Normal distribution was tested using the Kolmogorov–Smirnov test. For continuous variables, values are presented as the median and 5–95 percentiles. Differences between groups were determined using the U-Mann–Whitney test. Qualitative variables are shown as proportions compared using the Chi^2^ and Monte Carlo test when there was a zero value in any box. To determine the association between nutritional and physical parameters, a model of logistic regression adjusted by sex and age was used, reporting odds ratios (OR; with 95% CI). Values of *p* < 0.05 were considered statistically significant.

## 3. Results

### 3.1. Sociodemographic Characteristics of the Study Sample

Participants were a median of 31 years old (18–62 years old) among 172 men and 578 women and the highest percentage of the sample had university studies (61%), were single (56.7%), and belonged to a middle socioeconomic status (55.4%) (Table 1).

### 3.2. Physical Activity and Food Habits

Regarding anthropometric parameters, 35.86% of the sample reported being overweight and 16.43% reported obesity. Women had a higher prevalence of obesity than men (*p* = 0.03). The men reported a higher PA (times/week and min/session, *p* < 0.001) than women. Men also performed a higher number of active breaks than women (*p* = 0.04). The highest percentage of the sample passed ≥6 h sitting or sedentary (54.4%) (Table 2).

According to eating habits, the highest percentage of the study sample drank 3–5 glasses of water per day (45.3%) and consumed 1–2 portions of vegetables per day (69.1%), legumes 1–2 times per week (83.7%), 1–3 fruits per day (53.3%), and the same amount of vegetables as before (48.4%). Regarding meat consumption, 55.5% consumed red meat, 65.0% consumed white meat, and 75.1% consumed fish 1–2 times per week. Women reported cooking at home more frequently than men (*p* = 0.04). The majority of participants declared to have maintained their eating habits but in regard to consumption, the majority declared to eat more than before (51.3%). In relation to the negative parameters, 30% of the sample reported consuming alcohol daily and eating junk food and fried foods 1–2 times per week (62.9% and 59.9, respectively) (Table 3).

### 3.3. Body Weight Changes

**Figure 1:**
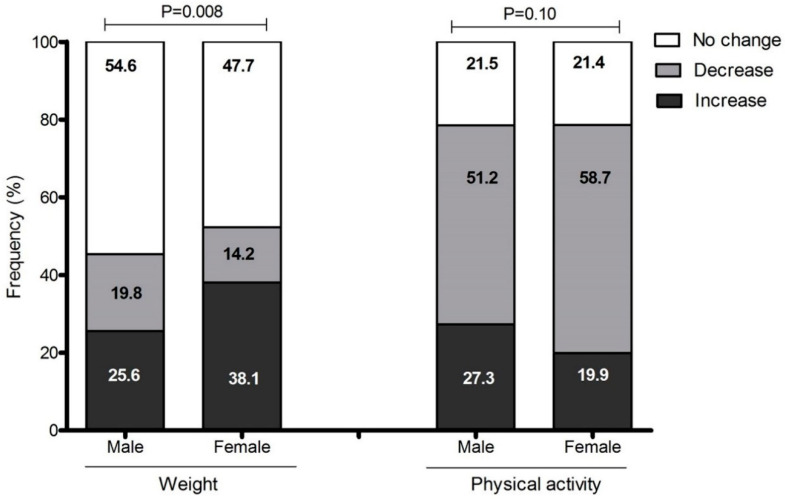
Perception of change in body weight and physical activity patterns during COVID-19 confinement.

Figure 1 shows the change in body weight and PA patterns. Of the men, 25.6% and of the women, 38.1% reported an increase in body weight (men vs. women, *p* = 0.008); 51.2% of the men and 58.7% of the women reported a decrease in PA levels (men vs. women, *p* = 0.10).

### 3.4. Association of Variables with Bodyweight Increase and PA Decrease

According to sociodemographic parameters, separated marital status presented the greatest association with body weight increase (OR; 3.33, 95% CI; 1.53–7.24, *p* = 0.002). Similarly, the middle socioeconomic level presented an association with body weight increase (OR; 1.48, 95% CI; 1.04–2.10, *p* = 0.027). In relation to foods habits, the consumption of fried foods ≥3 times per week (OR; 3.36, 95% CI; 1.77–6.4, *p* < 0.001), low water consumption (OR; 1.58, 95% CI; 1.03–2.41, *p* = 0.03), low consumption of legumes once per week (OR; 2.27, 95% CI; 1.05–4.92, *p* = 0.03), and junk food ≥ 3 times per week (OR; 1.76, 95% CI; 1.02–3.0, *p* = 0.04) had an association with body weight increase. Moreover, fish consumption presented an inverse association with body weight increase (OR; 0.67, 95% CI; 0.46–0.97, *p* = 0.03). Likewise, PA reported an inverse association with body weight increase, and potential protective factors stand out, including active breaks (OR; 0.72, 95% CI; 0.53–0.99, *p* = 0.04) and PA ≥ 4 times per week (OR; 0.51, 95% CI; 0.34–0.75, *p* = 0.001). Moreover, sedentary time ≥6 h/day had a positive association with body weight increase (OR; 1.85 95% CI; 1.13–3.03, *p* = 0.01) (Table 4).

Table 5 shows the variables associated with decreased PA. These included daily alcohol consumption (OR; 4.77, 95% CI; 1.68–13.5, *p* = 0.003), perception of body weight increase (OR; 2.01, 95% CI; 1.35–3.25, *p* = 0.001), and consumption of more food than before (OR; 1.87, 95% CI; 1.26–2.78, *p* = 0.002).

## 4. Discussion

The present study aimed to determine lifestyle changes, such as food habits and PA patterns (i.e., type of PA, time, duration, sedentary time), in the Chilean population during COVID-19 confinement and to analyze its association with changes in body weight and physical status. The main results of the present study were as follows: (a) low water consumption was associated with body weight increase; (b) active breaks may be protective factors for body weight increase; (c) daily alcohol consumption was associated with PA decrease; and (d) sedentary time ≥ 6 h/day presented an association with negative changes in body weight.

Negative eating habits, such as low consumption of legumes and water and high consumption of junk food (i.e., food with low food quality, low contribution of micronutrients and with a high contribution of sugar, saturated fat, and sodium) and fried foods, were associated with negative changes in body weight. In the case of legumes, the high content of dietary fiber, low energy density, high protein intake, and low glycemic index make them a food with high nutritional quality that enables the control of body weight and the prevention of metabolic disease [15,16]. The DGCP [14] recommended the consumption of legumes to be at least two times per week. However, recommendations of dietary guideline for the Spanish [8] and North American [17] populations suggest increasing the minimum consumption of legumes to three times per week in different types of presentation (i.e., salad, stews, sauces, soup, cream of legume’s, etc.) In this study, those evaluated had medium to low compliance with the general recommendation (83.7% indicated consuming 1–2 times a week) similar to that reported in another study in the North American population [18]. In contrast to our results, a recent study conducted in Spanish adults reported that the subjects increased consumption of foods, such as olive oil, vegetables, fruits, or legumes, during confinement. Moreover, this study reported a higher Mediterranean diet adherence (MDA) that could have a positive impact on the prevention of COVID-19-related complications [19].

In times of longer homestays and quarantine plans, changes occur in the daily routine and boredom is increased. This has been associated with a greater desire to consume pleasant foods to cope with the stress produced by confinement [20]. Junk food has a high proportion of refined sugars and saturated fats (in addition to frying), making consumption a risk factor for obesity and causing an increase in the pro-inflammatory state [21,22]. The DGCP recommended avoiding fried foods and foods with saturated fats, and to consume sugar sporadically and in small amounts. In this study, the consumption of junk food three times per week was associated with an increase in body weight and the study sample were not complying with DGCP recommendations [14]. Additionally, a recent study reported that food consumption and meal patterns were unhealthier during COVID-19 confinement [23]. Along this line, Bhutani et al. reported an increase in unhealthy foods and snacks during the COVID-19 home confinement [24].

Another important element that stands out as a protective factor in the increase of fat mass and favors the regulatory functions of hunger and satiety is the daily consumption of water [25]. In this case, the study sample presented a frequency of consumption of 3 to 4 glasses of water per day, with men reporting higher consumption than women (i.e., 4 glasses and 3 glasses, respectively), similar to that reported in other studies [26,27]. Water consumption of the sample remained under the recommendation of DGCP (recommendation of 6–8 glasses per day), which is highly related to a higher caloric intake [28].

For the control of cardiovascular diseases and body weight, the MDA has been one of the most accepted worldwide because it is characterized by a high concentration of antioxidants from legumes and vegetables and it contributes significantly with essential fatty acids from nuts and fish [29]. This report presented an inverse association between fish consumption and increased body weight, such as indicated by Jain et al. [30] However, this group had lower consumption in relation to the DGCP recommendations and the American Heart Association [31] (2 times a week) and compared to the Spanish population (2–3 times a week).

Active breaks and a different kind of PA are protective factors for body weight increase during COVID-19 confinement. Jakobsson et al. indicated that maintaining regular PA during self-isolation was important for prevention [32]. Moreover, the authors recommended interrupting sitting time with active breaks during the day. Conversely, lower PA levels increased the risk of gaining weight by reducing energy expenditure [33]. High levels of obesity increase the risk of infection and mortality in viral diseases, so it is advisable to avoid weight gain by including regular exercise [34]. A recent study of the Australian population indicated that 43.4% of the population (*n* = 5469) exercised less during the COVID-19 pandemic and found a relationship between binge eating and exercise [35]. Quarantine affected body weight increase (around 2.2–4.4 kg), indicating a decrease in PA to be one of the main risk factors [36]. Moreover, the perception of body weight increasing during confinement has been observed in 48.6% of the Italian population [37]. Therefore, it is essential to incorporate activities during the pandemic such as walking around the house, stair climbing, sitting and standing on a chair, raising PA levels, and increasing energy expenditure, thus avoiding body weight increase and mental health problems [38,39].

The findings of our study indicate that a sedentary time ≥6 h/day had a positive association with body weight increase; these results are opposite to those reported by Zachari et al. who found no relationship between sedentary time and body weight gain in times of quarantine [36]. Despite this, extended home quarantine may help generate body weight gain in adults [5]. Similarly, another study reported that COVID-19 home confinement had a negative effect on all PA intensity levels; moreover, the daily sitting time increased [23].

We found that a middle socioeconomic background was associated with body weight increase. Similarly, healthy lifestyle practices such as more favorable modifications of nutritional behaviors and PA levels during COVID-19 confinement are associated with higher incomes in the French population [13]. This could be related to greater financial resources and employment flexibility facilitating healthy choices. Conversely, Australian adults in the lowest income category had significantly higher mental health problems during COVID-19 compared to higher income adults [40]. Lifestyle (i.e., foods habits, PA) and mental health may affect body weight increase during COVID-19 confinement [13,19,23,40].

In the present study, 30% of the sample study reported daily alcohol consumption during confinement; in addition, this consumption was associated with negative changes in lifestyle, such as a decrease in PA. A negative change in alcohol intake was more likely to cause more depression, anxiety, and stress during confinement in the Australian population [40]. Evidence has shown that alcohol drinkers are less able to find anything positive about the pandemic situation and were mentally less able to cope [41]. Moreover, Jurak et al. suggested that being physically active is a simple and effective way of addressing the adverse effects of COVID-19 [42]. In contrast to our results, there is strong evidence for the existence of a positive association between alcohol consumption and PA [43,44,45]. Additionally, another study reported that moderate drinkers and heavy drinkers were more likely than abstainers to have physically active lifestyles in data representative of the U.S. population [46]. Piazza et al. reported that alcohol consumers were more physically active than non-drinking peers; moreover, the authors concluded that these findings were contrary to the hypothesis of the investigators [47]. Despite this, it has been demonstrated that excessive alcohol consumption is associated with an increased risk of mortality [48,49].

In this study, separated and married marital status was associated with body weight increase. One longitudinal study reported that changes in marital status, such as entering or leaving a marriage, influenced body weight; besides, women who were unmarried at baseline and married at follow-up had greater increase in body weight than women who were married at both times [50]. Moreover, Umberson et al. concluded that marital transitions are more important than marital status in predicting changes in body weight [51]. Future studies into COVID-19-related changes in behavior related to COVID-19 mitigation could ask about relationship history.

## 5. Strengths and Limitations

The main limitation of this study is its cross-sectional design; these factors should also be measured in a longitudinal study in the future to clarify the direction of the associations. Another limitation would be that the body weight and PA level were self-reported, which could mean that these data are underestimated or overestimated. Likewise, the results presented could have been regardless of the COVID-19 confinement. The main strength was that people from all over the Chilean national territory participated in this study and the study provides novel results applicable to confinement times.

## 6. Contributions of this Study

The present study provides different and relevant aspects that must be considered in times of COVID-19 confinement. Among them, water consumption and the development of active breaks can be recommended since they may be protective factors for body weight increase and they are simple factors to support and easy to apply for the population.

## 7. Conclusions

A healthy lifestyle that includes good food habits, PA, and active breaks are particularly important since they may be protective factors for body weight increase during COVID-19 confinement. Moreover, the evidence suggests that factors such as increasing water consumption and the performance of active breaks can be developed at the home as an easy way to avoid a body weight increase during COVID-19 confinement.

## Figures and Tables

**Table 1:** Sociodemographic characteristics of the study sample.

Variable	Total *n* = 700	Male *n* = 172 (24.6%)	Female *n* = 528 (75.4%)	*p*-Value
Age (years) ^a^	31 (18–62)	28 (18–61)	31 (19–62)	<0.001
Members per household (*n*) ^a^	3 (0.5–5.5)	2.5 (0–5)	3 (1–6)	0.179
Education level *n* (%) ^b^				0.189
Primary	5 (0.71)	0	5 (0.95)	
Secondary	92 (13.14)	26 (15.12)	66 (12.5)	
University	427 (61)	106 (61.63)	321 (60.8)	
Technical education	85 (12.14)	14 (8.14)	71 (13.45)	
Postgraduate	91 (13)	26 (15.12)	65 (12.31)	
Marital status *n* (%) ^b^				0.016
Single	396 (56.57)	116 (67.44)	280 (53.03)	
Married	185 (26.43)	35 (20.35)	150 (28.41)	
Common law married	73 (10.43)	14 (8.14)	59 (11.17)	
Separated	40 (5.71)	7 (4.07)	33 (6.25)	
Widowed	6 (0.86)	0	6 (1.14)	
Socioeconomic level ^b^				0.041
Low	47 (6.71)	10 (5.81)	37 (7.01)	
Middle–low	147 (21.00)	30 (17.44)	117 (22.16)	
Middle	388 (55.43)	93 (54.07)	295 (55.87)	
Middle–high	99 (14.14)	36 (20.93)	63 (11.93)	
High	19 (2.71)	3 (1.74)	16 (3.03)	

Note: Data shown represents numbers and proportions. *p* values < 0.05 are statistically significant. ^a^—represents median and 5 and 95 percentiles, ^b^—represents number and proportions.

**Table 2:** Anthropometric and physical characteristics of the study sample.

Variable	Total *n* = 700	Male *n* = 172 (24.6%)	Female *n* = 528 (75.4%)	*p*-Value
Anthropometric parameters				
Size (m) ^a^	1.63 (1.5–1.8)	1.75 (1.6–1.82)	1.6 (1.5–1.72)	<0.001
Body weight (kg) ^a^	68 (52–96)	76.5 (60–105)	66 (51–92)	<0.001
BMI (kg/m^2^) ^a^	25.3 (20.2–34.95)	25.85 (20.4–31.6)	25.1 (20.2–34.95)	0.720
BMI category n (%) ^b^				0.029
Normal weight	334 (47.71)	76 (44.19)	258 (48.86)	
Overweight	251 (35.86)	75 (43.6)	176 (33.3)	
Obesity	115 (16.43)	21 (12.2)	94 (17.8)	
Physical activity patterns				
Physical activity (times/week) ^a^	2 (0–7)	3 (0–7)	2 (0–7)	<0.001
Physical activity (min/session) ^a^	30 (0–90)	45 (0–120)	30 (0–90)	<0.001
Types of physical activity (%) ^b^				<0.001
None	198 (28.3)	30 (17.44)	168 (31.82)	
Combined/mixed	136 (19.43)	37 (21.51)	99 (18.75)	
Yoga/Pilates	77 (11)	12 (6.98)	65 (12.31)	
Aerobic/jogging	118 (16.86)	25 (14.53)	93 (17.61)	
Calisthenics	118 (16.86)	43 (25)	75 (14.2)	
Resistance training	53 (7.57)	25 (14.53)	28 (5.3)	
Review social network *n* (%Yes) ^b^	407 (58.14)	91 (52.9)	316 (59.85)	0.101
Active breaks *n* (%Yes) ^b^	285 (40.71)	86 (50.0)	199 (37.69)	0.004
Sedentary lifestyle *n* (Yes%) ^b^	407 (58.14)	91 (52.9)	316 (59.85)	0.100
Sedentary lifestyle (hours/day)				0.039
<2	85 (12.1)	17 (9.9)	68 (12.9)	
3–5	234 (33.4)	71 (41.3)	163 (30.9)	
≥6	381 (54.4)	84 (48.8)	297 (56.2)	

Note: Data shown represents numbers and proportions. *p* values < 0.05 are statistically significant. ^a^—represents median and 5 and 95 percentiles, ^b^—represents proportions. BMI—body mass index.

**Table 3:** Frequency of nutritional consumption in the study sample.

Variable	Total *n* = 700	Male *n* = 172 (24.6%)	Female *n* = 528 (75.4%)	*p*-Value
Positive Nutritional parameters				
Glasses of water drunk per day ^a^	3 (1–7)	4 (1–7)	3 (0–7)	0.016
Vitamin supplementation (yes %) ^b^	186 (26.6)	36 (20.9)	150 (28.4)	0.054
Glasses of water per day ^b^				0.041
0–2	138 (19.7)	41 (23.8)	97 (18.4)	
3–5	317 (45.3)	84 (48.9)	233 (44.1)	
6–7	245 (35)	47 (27.3)	198 (37.5)	
Vegetables per day (portions) ^b^				0.671
None	117 (16.7)	32 (18.6)	85 (16.1)	
1–2	484 (69.1)	118 (68.6)	366 (69.3)	
≥3	99 (14.1)	22 (12.8)	77 (14.6)	
Legumes, times per week ^b^				0.060
None	68 (9.7)	12 (7)	56 (10.6)	
1–2	586 (83.7)	143 (83.1)	443 (83.9)	
≥3	46 (6.7)	17 (9.9)	29 (5.5)	
Fruits per day (portion) ^b^				0.491
None	48 (6.9)	15 (8.7)	33 (6.3)	
1–3	373 (53.3)	92 (53.5)	281 (53.2)	
≥4	279 (39.9)	65 (37.8)	214 (40.5)	
Change consumption vegetables and fruits ^b^				0.301
Less than before	145 (20.7)	33 (19.2)	112 (21.2)	
Same than before	339 (48.4)	92 (53.5)	247 (46.8)	
More than before	216 (30.9)	47 (7.3)	169 (32)	
Red meat, time per week				0.018
None	122 (17.4)	20 (11.6)	102 (19.3)	
1–2	388 (55.5)	94 (54.7)	294 (55.7)	
≥3	190 (27.1)	58 (33.7)	132 (25)	
White meat, times per week ^b^				0.191
None	85 (12.1)	15 (8.7)	70 (13.3)	
1–2	455 (65.1)	112 (65.1)	343 (64.9)	
≥3	160 (22.9)	45 (26.2)	115 (21.8)	
Fish, times per week ^b^				0.590
None	150 (21.4)	36 (20.9)	114 (21.6)	
1 to 2	526 (75.1)	128 (74.4)	398 (75.4)	
≥3	24 (3.5)	8 (4.6)	16 (3.0)	
Dairy products, times per day ^b^				0.019
None	76 (10.9)	18 (10.5)	58 (11)	
1–2	530 (75.7)	120 (69.8)	410 (77.7)	
≥3	94 (13.4)	34 (19.8)	60 (11.4)	
Cook at home ^b^				0.004
Less than before	40 (5.7)	12 (7)	28 (5.3)	
Same than before	243 (34.7)	76 (44.2)	167 (31.6)	
More than before	417 (59.6)	84 (48.8)	333 (67.1)	
Feeding style perception				0.161
Less healthy than before	187 (26.7)	48 (27.9)	139 (26.3)	
Same than before	277 (39.6)	76 (44.19)	201 (38.1)	
Healthier than before	236 (33.7)	48 (27.9)	188 (35.6)	
Among of consumption food, perception				0.189
Less than before	104 (14.9)	32 (18.6)	72 (13.7)	
Same than before	237 (33.8)	60 (34.9)	177 (33.5)	
More than before	359 (51.3)	80 (46.5)	279 (52.8)	
Negative Nutritional parameters				
Alcohol ^b^				0.159
None	428 (61.1)	95 (55.2)	333 (63.1)	
1–3 times/day	210 (30)	61 (35.5)	149 (28.2)	
1–2 times/week	62 (8.9)	16 (9.3)	46 (8.7)	
Sausages, times per week ^b^				<0.001
None	285 (40.7)	44 (25.6)	241 (45.6)	
1–2	359 (51.3)	107 (62.2)	252 (47.7)	
≥3	56 (8)	21 (12.2)	35 (6.6)	
Junk food, times per week ^b^				0.038
None	175 (25)	50 (29)	125 (23.7)	
1–2	440 (62.9)	110 (64)	330 (62.5)	
≥3 times per week	85 (12.1)	12 (7)	73 (13.8)	
Fried food, times per week ^b^				0.149
None	223 (31.9)	47 (27.3)	176 (33.3)	
1–2	419 (59.9)	106 (61.6)	313 (59.3)	
≥3	58 (8.3)	19 (11.1)	39 (7.4)	

Note: Data shown represents numbers and proportions. *p* values < 0.05 are statistically significant. ^a^—represents median and 5 and 95 percentiles, ^b^—represents proportions.

**Table 4:** Factors related to the body weight increase perception in COVID-19 confinement.

Variable	OR (95% CI) *p*-Value
Sociodemographic parameters	
Separated marital status	3.33 (1.53–7.24), 0.002
Married	1.52 (1.03–2.2), 0.030
Middle socioeconomic level	1.48 (1.04–2.1), 0.027
Nutritional parameters	
Eating more food	4.12 (2.9–5.9), <0.001
Eating less food	4.12 (2.52–6.72), <0.001
Cook less than before	4.06 (1.9–8.7), <0.001
Eating fried foods ≥ 3 times a week	3.36 (1.77–6.4), <0.001
Perception of having a healthier diet	2.46 (1.72–3.54), <0.001
Low consumption of legumes ≤ 1 time per week	2.27 (1.05–4.92), 0.030
Sausage consumption ≥ 3 times per week	2.16 (1.18–3.97), 0.010
Junk food consumption ≥ 3 times per week	1.76 (1.02–3.0), 0.040
Low water consumption ≤ 2 glasses per day	1.58 (1.03–2.41), 0.030
Cook more than before	1.50 (1.1–2.1), 0.010
Fish consumption 1–2 times per week	0.67 (0.46–0.97), 0.030
Physical activity parameters	
Active breaks	0.72 (0.53–0.99), 0.040
Mixed physical activity	0.63 (0.40–0.99), 0.048
Exercise session duration 30–60 min	0.61 (0.42–0.90), 0.011
Yoga and Pilates	0.53 (0.31–0.90), 0.021
Exercise session duration > 60 min	0.52 (0.31–0.88), 0.010
Physical activity ≥ 4 times/week	0.51 (0.34–0.75), 0.001
Sedentary behavior ≥ 6 h/day	1.85 (1.13–3.03), 0.010

Note: The data show represent OR, (95% CI), *p*-value. The OR was adjusted by age and sex.

**Table 5:** Factors related to a decrease in physical activity in COVID-19 confinement.

Variable	OR (CI 95%) *p*-Value
Anthropometric parameters	
Perception of weight increase	2.01 (1.35–3.25), 0.001
Being Overweight	1.80 (1.17–2.76), 0.007
Nutritional parameters	
Daily alcohol consumption	4.77 (1.68–13.5), 0.003
Decrease vegetable consumption	3.32 (1.85–5.98), <0.001
Perception of having a healthier diet	2.11 (1.36–3.29), 0.001
Eating more food than before	1.87 (1.26–2.78), 0.002
Physical parameters	
Sedentary ≥ 6 h	2.12 (1.23–3.63), 0.006
Exercise session duration ≤ 30 min	1.99 (1.18–3.37), 0.01
Yoga and Pilates	1.82 (1.02–3.24), 0.04
Physical activity 1–3 times per week	1.67 (1.07–2.6), 0.02

Note: The data shown represents OR, (95% CI), *p*-value. OR was adjusted by age and sex.
